# CRS and HIPEC in patients with peritoneal metastasis secondary to colorectal cancer: The small-bowel PCI score as a predictor of survival

**DOI:** 10.1515/pp-2019-0018

**Published:** 2019-10-30

**Authors:** John Spiliotis, Vasileios Kalles, Ioannis Kyriazanos, Alexios Terra, Anastasia Prodromidou, Apostolos Raptis, Nikolaos Kopanakis, Athina Christopoulou

**Affiliations:** Department of Peritoneal Surface Oncology, Athens Medical Centre, Athens, Attica, Greece; Department of Surgery, Naval Hospital of Athens, Deinokratous 70, Athens, Greece; Department of Surgery, Metaxa Cancer Hospital of Piraeus, Piraeus, Attike, Greece; Department of Oncology, Saint Andreas General Hospital, Patras, Greece

**Keywords:** colorectal cancer, cytoreductive surgery, Hyperthermic IntraPeritoneal Chemotherapy (HIPEC), PCI score, small bowel

## Abstract

**Background:**

Combining cytoreductive surgery (CRS) with Hyperthermic IntraPeritoneal Chemotherapy (HIPEC) can benefit patients with peritoneal metastasis from colorectal cancer. The present study evaluates the small bowel subset of the Peritoneal Cancer Index (Small-Bowel-PCI score (SB-PCI), min-max 0–12) as a prognostic factor in such patients.

**Methods:**

We retrospectively analyzed patients that underwent CRS and HIPEC for recurrent colorectal cancer with peritoneal metastasis. Patient characteristics, procedure details, and clinical outcomes were evaluated.

**Results:**

Eighty patients were included. The mean intraoperative PCI-score was 16.8, with a mean SB-PCI score of 5.9. CC0/1 was achieved in 62/80 patients. The mean follow-up period was 26.3 months. Univariate regression analysis showed that the ECOG status, the presence of severe complications, the HIPEC regimen (oxaliplatin vs. mitomycin-C), the PCI score, the SB-PCI score and the completeness of cytoreduction correlated significantly with overall survival. In multivariate analysis, the SB-PCI and CC score were identified as independent prognostic factors of survival. When the SB-PCI was stratified in three groups (0–4, 5–8 and 9–12), Kaplan–Meier curve analysis showed significant difference in survival (p<0.001).

**Conclusions:**

The SB-PCI correlates with overall survival in patients with peritoneal metastases secondary to colorectal cancer in this retrospective cohort. Its use should be validated in prospective patient series.

## Introduction

Colorectal cancer represents the third most common cancer and the fourth most common cause of cancer-related death worldwide [1]. The peritoneum is the second most frequent site of colon cancer metastasis, and approximately 4–7% of newly diagnosed patients with colon cancer are found to have peritoneal dissemination of the disease, despite the recent advances that facilitate early detection of the disease [2, 3]. Peritoneal metastasis from colorectal cancer origin has been associated with poor prognosis as well as poor quality of life for the patients in this terminal stage of the disease [3, 4]. Therefore, patients with colorectal cancer peritoneal metastasis have been regarded as terminal, with only palliative surgery and/or systemic chemotherapy being recommended [5].

Recently, in selected patients, the combination of extensive cytoreductive surgery (CRS) with Hyperthermic intra-operative IntraPeritoneal Chemotherapy (HIPEC) has been reported to confer a benefit in survival of patients with peritoneal metastasis from colorectal cancer [6, 7, 8]. The first randomized trial comparing cytoreduction plus HIPEC followed by systemic chemotherapy vs. systemic chemotherapy only, showed significant benefit from the combined treatment [7].

Among the factors that are associated with prognosis in colorectal cancer patients with peritoneal metastasis undergoing CRS and HIPEC, despite the variations in chemotherapy regimens and techniques of hyperthermia [9], the most important ones that appear consistently in the literature are the completeness of cytoreduction and the extent of the disease, as measured using the peritoneal cancer index (PCI) [10, 11, 12]. Therefore, it is now considered that only patients with PC below a certain threshold undergoing good-quality cytoreductive surgery will benefit from CRS and HIPEC.

In this context, the present study aims to evaluate the prognostic impact of the small bowel subset of the PCI (SB-PCI) score in patients with PC secondary to colorectal cancer.

## Materials and methods

The present study is a retrospective analysis of prospectively maintained data from patients that underwent cytoreductive surgery followed by HIPEC for resectable peritoneal metastasis from colorectal cancer in the two participating institutes between 2010 and 2017. The study was approved by both Institutional Ethics and Research Boards.

### Patient selection

All patients underwent detailed preoperative assessment including radiological and/or laparoscopic staging to estimate the extent of peritoneal dissemination and resectability of the disease. Radiological staging included thoracic, abdominal and pelvic computed tomography (CT) with oral and intravenous contrast, and/or abdominal magnetic resonance imaging (MRI). The patient’s performance status was assessed based on the Eastern Cooperative Oncology Group (ECOG) scale [13]. All cases were discussed in a dedicated Multi-Disciplinary Team (MDT) meeting, in which treatment options were discussed, and the MDT results were available to the patients before surgery.

### Procedure details

In all cases, a laparotomy was performed and the extent of peritoneal disease was calculated intraoperatively using the PCI score, as described by Sugarbaker et al. [10]. In case the tumor burden was deemed to be resectable, cytoreduction was performed using tumor removal, organ resections and peritonectomy techniques as described by Sugarbaker et al. [10] After the surgical procedure, the completeness of cytoreduction (CC) was evaluated for each patient as follows: a CC-0 score indicated no visible tumor in the peritoneal cavity; a CC-1 score indicated residual tumor <2.5 mm; a CC-2 score indicated residual tumor 2.5–2.5 cm; a CC-3 score indicated a residual tumor >2.5 cm [10]. Patients with CC-0/CC-1 scores were considered to have undergone complete cytoreduction.

Following cytoreduction, patients underwent HIPEC using the closed abdomen technique. During the closed abdomen technique, all intestinal reconstructions are performed before closure of the abdomen, four tubes (two for inflow of the chemotherapy solution and two for outflow) are inserted and the abdomen was closed with standard abdominal closure techniques. After testing for possible leaks with normal saline 0.9%, mitomycin (MMC) or oxaliplatin was administrated in the abdominal cavity at an intraperitoneal temperature of 42 °C at a dose of 15 mg/m^2^ (MMC) or 360 mg/m^2^ (oxaliplatin) for 60–90 min.

### Parameters evaluated

For each patient, demographic data (age, sex, ECOG status), details of the course of the disease (site of primary tumor, previous chemotherapy, previous administration of bevacizumab), and procedural details (PCI score, length of surgery in minutes, CC score, HIPEC regimen) were recorded. The small bowel subset of the PCI score (Small Bowel-PCI score (SB-PCI), min-max 0–12) was recorded separately for each patient. As far as the postoperative course was concerned, the length of ICU stay as well as the length of hospital stay was recorded, while postoperative morbidity was graded according to the Clavien-Dindo classification system [14]. Overall survival was defined from the time of the surgical procedure to the date of reported death, and disease-free survival was defined from the time of the surgical procedure to the time of diagnosis of disease recurrence or progression.

### Statistical analysis

Overall survival was used as the primary endpoint of this study. For categorical variables, the chi-square and Fisher’s exact test were used as appropriate. Survival analysis was performed using the Kaplan–Meier method, and compared using the log-rank test. Multivariate analyses using Cox-regression models (Forward LR and Backward LR) were performed in order to identify independent prognostic factors of survival.

A p-value of less than 0.05 was considered statistically significant and the analysis was performed using SPSS v 20 for Windows (SPSS, Chicago, IL, USA).

## Results

Eighty patients (44 males – 36 females) with a mean age of 57 years (range 33–75) years were included in the analysis. The mean intraoperative PCI score was 16.8 (range 1–39) with a mean small bowel PCI score of 5.9 (range 0–12). The mean operative time was 241 min (140–510 min). The patient sample characteristics are summarized in Table 1.

**Table 1:** Patient sample characteristics.

	n (%)
Number of patients	80
Men, n	44 (55%)
Women, n	36 (45%)
Mean age, years (range)	51 (33–75)
ECOG status	
0	14 (17.5%)
1	54 (67.5%)
2	8 (10%)
3	3 (3.7%)
4	1 (1.3%)
Preoperative chemo	
Yes	67 (83.7%)
No	13 (16.3%)
Preoperative administration of bevacizumab	
Yes	30 (37.5%)
No	50 (62.5%)

Complete cytoreduction (CC0/1) was achieved in 62/80 patients (77.5%), with 44/80 patients scored as CC-0 and 18/80 patients scored as CC-1. The intraoperative PCI score was significantly lower in patients in whom complete cytoreduction was achieved: patients with CC-0/1 cytoreduction had a median PCI score of 12 (range 3–28) whereas patients with CC-2/3 cytoreduction had a mean PCI score of 30 (range 14–39) (Mann-Whitney U test, p<0.001). Furthermore, 30/80 patients (37.5%) required postoperative ICU admission and the mean length of postoperative stay in the ICU was 11.6 days. Severe complications (Clavien-Dindo III/IV) were encountered in 20% of the cases whereas the 30-day mortality rate was 2.5%. The most frequently encountered complication was pleural effusion requiring drainage (9 cases, 11.25%) There were no re-admissions related to the procedure within 30 days from the operation.

**Figure 1:**
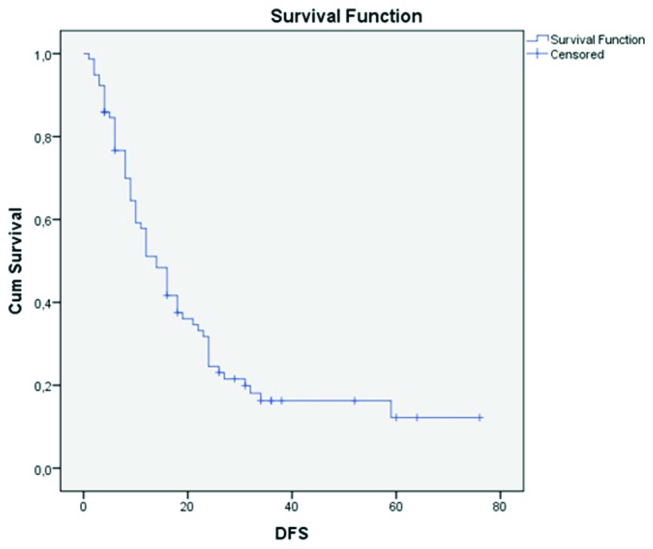
Kaplan–Meier curve: disease-free survival in our patient sample.

**Figure 2:**
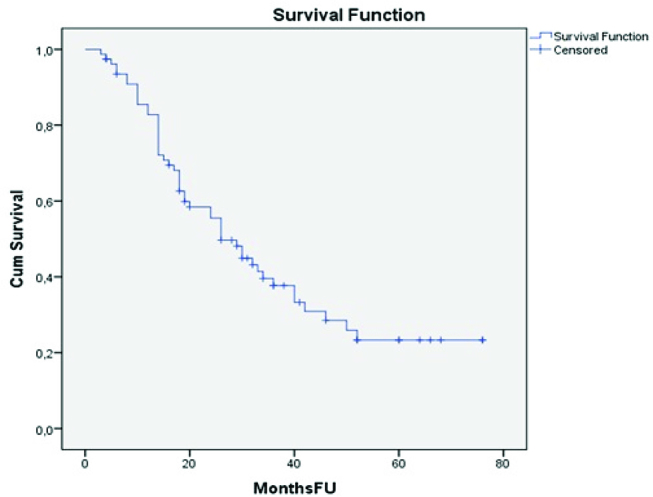
Kaplan–Meier curve: overall survival in our patient sample.

The mean follow-up period was 26.3 months. The median disease-free survival was 14 months (Figure 1) whereas the median overall survival was 26 months (Figure 2). Univariate regression analysis showed that the ECOG status, the presence of severe complications, the HIPEC regimen, the PCI score, the SB-PCI score, and the completeness of cytoreduction (CC score), were all parameters that correlated significantly with overall survival (Table 2).

**Table 2:** Univariate analysis, parameters affecting overall survival.

	p
Age	NS
Previous chemotherapy	NS
Site of primary tumor (Colon vs. rectum)	NS
ECOG status	p=0.014 (OR 3.6, 95% CI: 1.7–10.2)
Presence of severe complications (Clavien-Dindo III/IV)	p<0.001 (OR 3.1, 95% CI: 1.7–5.8)
HIPEC regimen (MMC vs. oxaliplatin)	p=0.001 (OR 2.6, 95% CI: 1.5–2.7)
PCI score	p<0.01 (OR 1.18, 95% CI: 1.13–1.23)
SB-PCI score	p<0.001 (OR 1.4, 95% CI: 1.28–1.53)
SB-PCI score category	p<0.001 (OR 4.32, 95% CI: 2.94–6.34)
Completeness of cytoreduction	p<0.001 (OR 4.29, 95% CI: 2.99–6.15)

**Figure 3:**
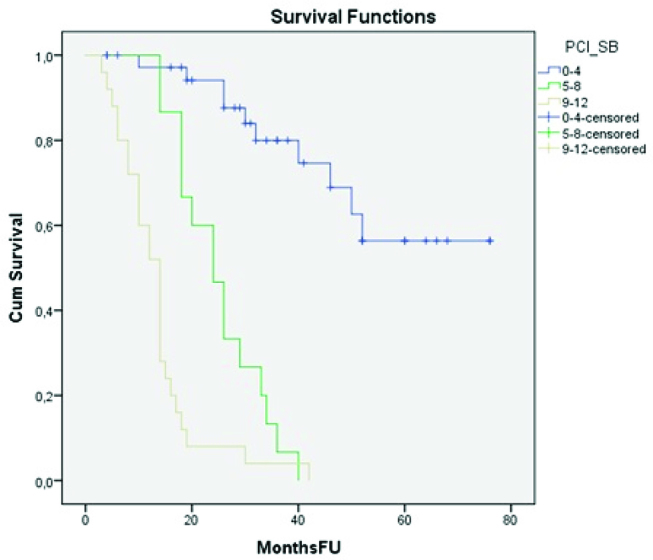
Kaplan–Meier curves: overall survival according to SB-PCI score category.

Kaplan–Meier curve analysis showed that, when the SB-PCI was stratified in three groups (0–4, 5–8 and 9–12), there was a statistically significant difference in survival between the three groups (log-rank test, p<0.001) (Figure 3, Table 3). Notably, all patients in the 0–4 and 5–8 SB-PCI groups had complete cytoreduction (CC-0/1), whereas only 33% of the patients in the 9–12 SB-PCI group were scored as CC-0/1. Further statistical analysis showed a significant correlation between the SB-PCI score and the completeness of cytoreduction (Spearman’s rho 0.870, p<0.001). Kaplan–Meier curve analysis also showed a significant difference in median survival (18 months vs. 42 months, p=0.001) between patients in whom oxaliplatin was administered vs. MMC.

**Table 3:** Overall survival analysis according to SB-PCI category.

SB-PCI score category	Number of patients	OS (mean)	OS (median)	p
0–4	38	58.9 (95% CI 50.5–67.3)	Not applicable	<0.001
5–8	15	24.9 (95% CI 20.8–29.0)	24 (18.3–29.7)	
9–12	25	13.4 (95% CI 10.1–16.6)	14 (11.8–16.2)	

When entered in a multivariate regression model, the SB-PCI score (p=0.007) and the CC score (p=0.025) were identified as independent prognostic factors of survival, adjusted for all covariates (age, sex, ECOG status, HIPEC regimen and presence of severe complications), whereas the HIPEC regimen was marginally not significantly associated with overall survival as an independent factor (Table 4).

**Table 4:** Multivariate analysis, independent prognostic factors of overall survival.

	p
Age	NS
Sex	NS
ECOG status	NS
Presence of severe complications (Clavien-Dindo III/IV)	NS
HIPEC regimen (MMC vs. oxaliplatin)	NS (p=0.09)
SB-PCI score	p=0.007
Completeness of cytoreduction	p=0.025

## Discussion

Peritoneal metastasis has historically been considered a terminal incurable disease. The introduction of CRS, which essentially consists of removal of all macroscopic disease using certain standardized surgical techniques, with the addition of HIPEC in order to eradicate any residual tumor burden, has changed the management of these patients, conferring significant survival benefit. In selected cases of patients with peritoneal metastasis secondary to colorectal cancer, a median survival of up to 63 months has been reported [6, 15, 16, 17]. In the present study, we report a median overall survival of 35, 6 months, which is in line with OS previously reported, and supports the effectiveness of the technique.

During the last decade, a lot of research has focused on the establishment prognostic factors of outcome in patients undergoing CRS and HIPEC, in order to optimize selection of patients who will benefit more from this aggressive surgical approach. Among the prognostic factors, completeness of cytoreduction (CC-0/1) has been shown to be the most important prognostic indicator, as 5-year survival in patients undergoing a CC-0 cytoreduction has been reported to be higher compared to that of patients undergoing CC-1 or CC-2 resections [16].

The volume of peritoneal metastasis present prior to cytoreduction remains another significant prognostic factor, as estimated using the PCI score. The relationship between the PCI score and survival in these patients has become more solid, as more data become available; however the specific cutoff point associated with poor prognosis has yet to be defined, with the limit currently being between 10 and 20 [18, 19, 20]. Sugarbaker et al. initially reported significantly higher survival (41 months) in patients with a PCI score less than 20, compared to a mere 16 month for patients with a PCI score greater than 20 [21]. Yan and Morris [19] later showed that a PCI score ≤10 was a significant favorable prognostic factor, whereas recently Faron et al., using a linear statistical model to characterize the relationship between the PCI score and survival concluded that the hazard ratio for each PCI score point increase is equal to [11].

The PCI score itself provides an excellent estimate of the extent of peritoneal metastasis, as it uses both the distribution and the volume of the disease, to form a numerical score for the extent of the tumor burden in a uniform way. However, as the numerical score often is being used as quantitative data in the interpretation of results, our study aimed to examine the prognostic importance of a specific component of the PCI score, the small bowel PCI score.

It is well known that the involvement of the small bowel may be a significant cause of incomplete cytoreduction, especially as the disease is often located at the verge between the mesentery and the small bowel, where the terminal arteries of the mesentery enter the bowel wall [22]. In a previously published study, the authors report that the involvement of area 10 (distal jejunum) was identified as an independent prognostic factor of survival [9]. The authors comment that although the involvement of the rest of the small bowel did not emerge as a prognostic indicator, this may have been due to lack of statistical power.

In the present study, our analysis confirms that the small bowel involvement remains a significant prognostic indicator of survival in patients undergoing CRS and HIPEC for peritoneal metastasis secondary to colorectal cancer. Moreover, it seems that there is significant difference in the survival of patients according to the extent of tumor burden in the small bowel (SB-PCI scores 0–4, 5–8 and 9–12), and that the presence of extensive small bowel involvement is associated more frequently with a CC-2 resection. In the clinical setting, this means that patients with extensive small bowel involvement are probably more likely to have a higher PCI score, and more likely to have a CC-2 cytoreduction, and therefore have a poor outcome.

One of the findings of the present study that should not be overlooked is the difference observed in overall survival according to the HIPEC regimen (MMC vs. oxaliplatin). Although the HIPEC regimen did not prove to be a significant independent prognostic factor in our patient sample, it comes in an era that the recently presented results of the PRODIGE 7 trial showed no benefit in overall survival for patients that received cytoreductive surgery combined with HIPEC using oxaliplatin vs. cytoreductive surgery alone. As one of the main criticisms on the study is the choice of the HIPEC regimen, it is evident that the comparison of survival outcomes between MMC and oxaliplatin merits further investigation.

The present study was designed in order to investigate the SB-PCI score as a prognostic factor of survival in patients undergoing CRS and HIPEC for PM secondary to colorectal cancer. The study benefits form a relatively homogenous patient sample, the fact that the data come from a prospectively maintained database, and the high standardization of the surgical technique and clinical practice between the two centers that contributed to the study. Still, our patient sample is relatively small, and our results ought to be confirmed in larger future studies. Another possible limitation of the study comes from the fact that the vast majority of the patients were referred for treatment to our center having already their imaging staging done elsewhere. As a result, uniform reporting of preoperative imaging studies was not feasible and therefore we are not able to report differences in the preoperative vs. intraoperative PCI or SB-PCI score.

In this context, it is important to highlight that preoperative staging by means of radiological assessment remains an important issue in patients undergoing CRS and HIPEC [23]. Although CT has historically been the most widely used modality, it seems to consistently underestimate the PCI score, and its sensitivity for the four small bowel and mesenteric sites has been reported to be as low as 21–25% [24]. MRI has been found to be able to predict the surgical PCI more accurately in these regions; however its use is often hampered by practical difficulties (e. g. low availability or long waiting times) and patient-related contraindications [25].

In conclusion, our results indicate that the SB-PCI correlates significantly with overall survival in patients with peritoneal metastases secondary to colorectal cancer, and its use should be further investigated in larger patient series.
